# Changes in T-Cell and Monocyte Phenotypes *In Vitro* by *Schistosoma mansoni* Antigens in Cutaneous Leishmaniasis Patients

**DOI:** 10.1155/2012/520308

**Published:** 2012-11-13

**Authors:** Aline Michelle Barbosa Bafica, Luciana Santos Cardoso, Sérgio Costa Oliveira, Alex Loukas, Alfredo Góes, Ricardo Riccio Oliveira, Edgar M. Carvalho, Maria Ilma Araujo

**Affiliations:** ^1^Serviço de Imunologia, Complexo Hospitalar Universitário Professor Edgard Santos, Universidade Federal da Bahia, 5 Rua João das Botas s/n, Canela, 40110-160 Salvador, BA, Brazil; ^2^Departamento de Ciências da Vida, Universidade do Estado da Bahia, 2555 Rua Silveira Martins, Cabula, 41.150-000 Salvador, BA, Brazil; ^3^Departamento de Bioquímica e Imunologia, Instituto de Ciências Biológicas, Universidade Federal de Minas Gerais, 6627 Avenida Antônio Carlos, Pampulha, 31270-901 Belo Horizonte, MG, Brazil; ^4^Instituto Nacional de Ciência e Tecnologia em Doenças Tropicais (INCT-DT-CNPQ/MCT), Rua João das Botas s/n, Canela, 40110-160 Salvador, BA, Brazil; ^5^Queensland Tropical Health Alliance and School of Public Health and Tropical Medicine, James Cook University, Cairns, QLD 4878, Australia; ^6^Escola Bahiana de Medicina e Saúde Pública, No. 275 Avenida Dom João VI, Brotas, 40290-000 Salvador, BA, Brazil

## Abstract

High levels of proinflammatory cytokines such as IFN-**γ** and TNF are associated with tissue lesions in cutaneous leishmaniasis (CL). We previously demonstrated that *Schistosoma mansoni* antigens downmodulate the *in vitro* cytokine response in CL. In the current study we evaluated whether *S. mansoni* antigens alter monocyte and T-lymphocyte phenotypes in leishmaniasis. Peripheral blood mononuclear cells of CL patients were cultured with *L. braziliensis* antigen in the presence or absence of the *S. mansoni* antigens rSm29, rSmTSP-2- and PIII. Cells were stained with fluorochrome conjugated antibodies and analyzed by flow cytometry. The addition of rSm29 to the cultures decreased the expression of HLA-DR in nonclassical (CD14^+^CD16^++^) monocytes, while the addition of PIII diminished the expression of this molecule in classical (CD14^++^CD16^−^) and intermediate (CD14^++^CD16^+^) monocytes. The addition of PIII and rSmTSP-2 resulted in downmodulation of CD80 expression in nonclassical and CD86 expression in intermediate monocytes, respectively. These two antigens increased the expression of CTLA-4 in CD4^+^ T cells and they also expanded the frequency of CD4^+^CD25^high^Foxp3^+^ T cells. Taken together, we show that *S. mansoni* antigens, mainly rSmTSP-2 and PIII, are able to decrease the activation status of monocytes and also to upregulate the expression of modulatory molecules in T lymphocytes.

## 1. Introduction

American tegumentary leishmaniasis is a disease caused by parasites of the genus *Leishmania*. This disease represents a significative public health problem worldwide with an incidence of 1.5 million new cases in recent years [1]. Tegumentary leishmaniasis presents with a wide spectrum of clinical manifestations ranging from localized skin to widespread mucocutaneous lesions depending on the parasite species [2] and the host immune response [3, 4]. T cell-mediated immunity is crucial to host protection against *Leishmania* sp. infection; however skin and mucosal lesions occurs due to a deregulated T helper 1 (Th1) cell response with high production of proinflammatory cytokines such as IFN-*γ* and TNF [3, 4]. 

Experimental studies have shown that *Schistosoma mansoni* infection or parasite products, by inducing regulatory cells and cytokines, are able to prevent some Th1-mediated autoimmune diseases in mice such as type I diabetes, experimental autoimmune encephalomyelitis, and psoriasis [5–7]. Recently, we demonstrated that the recombinant *S. mansoni* antigens Sm29 SmTSP-2, and also PIII down-modulated the production of IFN-*γ* and TNF in a group of cutaneous leishmaniasis (CL) patients [8]. In the present study, these antigens were tested regarding their ability to alter the monocyte and lymphocyte profiles. Studies have shown that Sm29 and SmTSP-2 antigens are secreted by the membrane and/or tegument of the *S. mansoni* adult worm. Proteins secreted or localized on the surface of *Schistosoma *spp., which are in intimate contact with host tissues, might be more effective in triggering immunoregulatory processes [9]. The Sm29 is a membrane-bound glycoprotein located on the tegument of the adult worm and lung stage schistosomula [10]. SmTSP-2 is a recombinant protein (tetraspanin) from *S. mansoni* tegument. In a mice model, immunization with SmTSP-2 resulted in a 57% reduction in adult worm burdens and a 64% reduction in liver egg burdens compared with control animals [11]. PIII is a multivalent antigen obtained from *S. mansoni *adult worms that modulates granuloma size in mice infected with the parasite [12, 13]. These antigens have been evaluated by our group regarding their potential to induce IL-10 production and suppress Th2 response *in vitro* in cells of asthmatic individuals [14]. 

Together with the Th1 immune response, monocytes and macrophage are key cells in controlling *Leishmania *sp. infection. Monocytes have been classifying into different subpopulations in mice models and also in humans. A new nomenclature was recently published defining human monocytes into three subtypes. The major population of human monocytes (90%) presents high expression of CD14 and lack of expression of CD16 (CD14^++^CD16^−^). They are referred to as classical monocytes. Intermediate monocytes are those which express CD14 and low CD16 expression (CD14^++^CD16^+^), while the nonclassical monocytes express CD16 and relatively low CD14 (CD14^+^CD16^++^) [15]. In a study conducted by Wong et al. [16] the characteristics of classical, intermediate and, nonclassical monocytes were evaluated through the gene expression profiling. They observed that classical monocytes express genes involved in angiogenesis, wound healing, and coagulation, being involved in tissue repair functions in addition to high expression of proinflammatory genes [16]; intermediate monocytes highly express MHC class II indicating they have antigen presenting cell function and T cell stimulatory properties. Nonclassical monocytes express genes involved in cytoskeleton rearrangement, which may be responsible for its high motility observed *in vivo* [17]. 

A high frequency of CD16^+^ monocyte subsets have been demonstrated in *Mycobacterium tuberculosis* infection and in viral infections such as hepatitis B (HBV) [18], hepatitis C (HCV) [19], HIV [20], and Dengue [21]. In CL patients, the frequency of CD14^+^CD16^+^ monocytes was significantly higher compared to healthy controls and they were positively correlated with the lesion size [22]. This subtype of monocytes is considered as antigen presenting cell [16], which produces cytokines and might be the most important subtype in activating T cells response.

The activation of T cells requires the antigen recognition in the MHC context and also signaling given by co-stimulatory molecules which interact with corresponding ligands on antigen presenting cells (APC). One of the most important co-stimulatory molecules on T cells is CD28, which is constitutively expressed and binds to CD80 and CD86 on the APC. CD86 is constitutively expressed at low levels and it is rapidly upregulated after primary antigen recognition, whereas CD80 exhibits delayed expression kinetics [23]. Other ligand for CD80 and CD86 is CTLA-4. The expression of this molecule is rapidly upregulated after T cell activation and provides a negative signal limiting the immune response [24–26]. *In vitro* study has shown that the addition of CTLA-4-Ig to block CD28-B7 interaction in CL patients PBMC cultures stimulated with *Leishmania* antigen led to a downmodulation of IFN-*γ* and TNF secretion [27]. Other similar study showed that blocking the co-stimulatory molecules CD80 and CD86 in human macrophages from *Leishmania*-naive donors infected with *L. major* resulted in a significant reduction in IFN-*γ*, IL-5, and IL-12 production [28].

In this study we evaluated whether *S. mansoni* antigens alter the expression of CD80 and CD86 on monocytes of CL patients and also the expression of CTLA-4 in T lymphocytes. Additionally, we accessed the frequency of regulatory T cells induced by *S. mansoni *antigens when they were added to the cell cultures stimulated with* Leishmania* antigens. 

The CD4^+^CD25^+^ regulatory T cells have been extensively studied due to their critical function in maintaining self-tolerance. These cells can express both low and high CD25 levels in mice models; however, only the CD4^+^CD25^high^ population exhibits a strong regulatory function in humans. These CD4^+^CD25^high^ T cells also express FOXP3, a molecule associated with regulatory functions [29]. This T cell subset in humans comprises ~1.5–3% of circulating CD4^+^ T cells. They inhibit proliferation and cytokine secretion induced by TCR cross-linking of CD4^+^CD25^−^ responder T cells in a contact-dependent manner [30] and completely abrogate IL-2-dependent proliferation of NK cells [31]. Moreover, regulatory T cells are able to downregulate the intensity and duration of both Th1 and Th2 immune responses in infectious diseases limiting damage to self-tissue [32, 33].

Our hypothesis in the present study is that the pathology of cutaneous leishmaniasis results from monocyte and T cell hypersensitivity due to impaired regulatory mechanisms. In this context, the use of *S. mansoni* antigens which induce regulatory cells and molecules would prevent the inflammatory process. To test this hypothesis we evaluated whether the addition of *S. mansoni* antigens to cell cultures of leishmaniasis patients would modify the phenotype and activation status of monocytes and lymphocytes. Specifically, we evaluated the expression of HLA-DR, CD80, and CD86 on classical, intermediate, and nonclassical monocytes and CD28, CTLA-4, CD25, and Foxp3 in T lymphocytes from CL patients in response to the soluble *Leishmania braziliensis* antigen (SLA) in the presence or absence of the *S. mansoni* antigens rSm29, rSmTSP-2, and PIII. 

## 2. Materials and Methods 

### 2.1. Patients and the Endemic Area

The study included the first 30 cutaneous leishmaniasis patients living in the endemic area of tegumentary leishmaniasis, Corte de Pedra, Bahia, Brazil, who attended the local Health Post from March 2010 to March 2012 and agreed in participate. Corte de Pedra is located in the southeast region of the State of Bahia, Brazil which is well known for its high rate of *L. braziliensis *transmission. 

The diagnostic of cutaneous leishmaniasis was made considering a clinical picture characteristic of CL, parasite isolation or a positive delayed-type hypersensitivity (DTH) response to *Leishmania* antigen, and a histological feature of CL. 

The inclusion criteria to this study were patients with 5 to 60 years of age diagnosis of CL with the presence of active skin lesions. The exclusion criteria were pregnancy, chronic diseases such as diabetes and asthma, and also HIV and HTLV-1 infection. 

Immunological analyses were performed prior to the specific therapy to leishmaniasis for all patients. There were not enough cells to perform the whole experiment every time since they require a larger number of cells more than what could be obtained from some patients. 

The frequency of helminth infection in the leishmaniasis endemic area of Corte de Pedra is 88.3% and *S. mansoni* infection presents in 16.7% of cutaneous leishmaniasis patients. 

The Ethical Committee of the Maternidade Climério de Oliveira, Federal University of Bahia approved the present study, and an informed consent was obtained from all participants or their legal guardians.

### 2.2. Antigen Preparation

The soluble *Leishmania* antigen used in this study was obtained from a* Leishmania* lysate (crude antigen). It was prepared from a *L. braziliensis* strain (MHOM/BR/2001) as previously described [34]. 

The *S. mansoni* antigens used in this study included Sm29, a *Schistosoma mansoni* recombinant protein [10]; SmTSP-2, a recombinant protein (tetraspanin) from *S. mansoni* tegument [11]; a fraction of *S. mansoni *soluble adult worm antigen (SWAP) obtained by anionic chromatography (FPLC), called PIII. The Sm29 was cloned in *E. coli *and tested for lipopolysaccharide (LPS) contamination using a commercially available LAL Chromogenic Kit (CAMBREX). The levels of LPS in Sm29 were below 0.25 ng/mL. However in order to neutralize potential effects of LPS presented in low levels in the *S. mansoni* recombinant antigen, Polymyxin B was added to cell cultures every 12 hours according to the established protocol [35]. The SmTSP-2 used in this study was produced in *Pichia pastoris* fermentation cultures and it has been kindly provided by Dr. Alex Loukas [11, 36]. The proteins rSm29 and PIII were provided by the Institute of Biological Science, Department of Biochemistry and Immunology, UFMG, Brazil.

### 2.3. Cell Culture and Intracellular and Surface Staining

Intracellular and surface molecules were evaluated by flow cytometry (FACSort, BD Biosciences, San Jose, CA). In order to perform the intracellular staining, peripheral blood mononuclear cells (PBMC, 3 × 10^5^) obtained through the Ficoll-Hypaque gradient were cultured *in vitro* with SLA (5 *μ*g/mL) in the presence or absence of the recombinant *S. mansoni* antigens Sm29, SmTSP-2, and PIII (5 *μ*g/mL) for 20 h, 37°C, and 5% of CO_2_. During the last 4 h of culture, Brefeldin A (10 *μ*g/mL; Sigma, St. Louis, MO), which impairs protein secretion by the Golgi complex, was added to the cultures. Afterwards, the cells were washed in PBS and fixed in 4% formaldehyde for 20 min at room temperature. Specific staining was performed with cychrome (CY)-labeled antibody conjugated with anti-CTLA-4 mAbs in saponin buffer (PBS, supplemented with 0.5% BSA and 0.5% saponin) and phycoerythrin (PE)-labeled antibody conjugated with anti-Foxp3 in Foxp3 staining buffer set (eBioscience). For double or triple staining, mAbs to human CD4, CD8, and/or CD25 conjugated with FITC or CY were used.

The evaluation of co-stimulatory molecule expression was performed in PBMCs stimulated for 60 h with the same antigens aforementioned. Cells were incubated with fluorescein isothiocyanate (FITC)-, PE-, or CY-labeled antibody solutions for 20 min at 4°C in a volume of 20 *μ*L in PBS. After staining, preparations were washed with 0.1% sodium azide PBS, fixed with 200 *μ*L of 4% formaldehyde in PBS and kept at 4°C. The antibodies used for the staining were immunoglobulin isotype controls-FITC, PE and CY, anti-CD4-FITC, anti-CD8-FITC, anti-CD25-FITC, anti-CD14-FITC, CD16-CY, anti-CD4-CY and anti-CD8-CY, anti-CD80-PE, anti-CD86-PE, anti-HLA-DR-PE, anti-CD28-PE (all from BD Biosciences Pharmingen). A total of 50,000 and 100,000 events were acquired for surface and intracellular experiments, respectively.

### 2.4. Analysis of FACS Data

**Figure 1:**
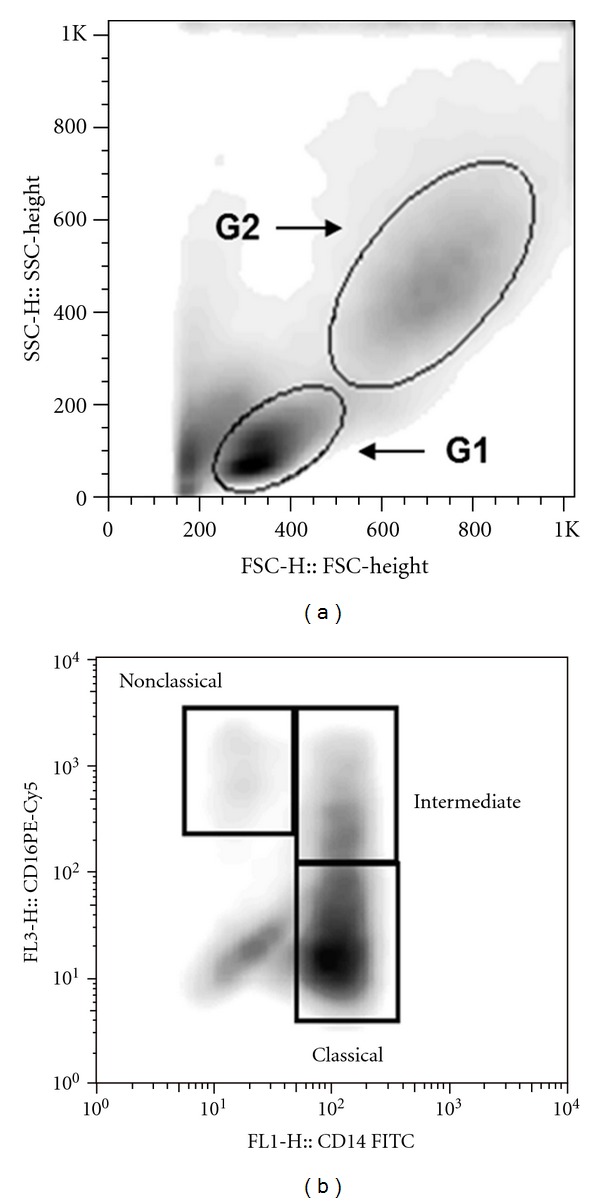
Strategy for T cell and monocytes evaluation by flow cytometry. The cell populations were defined by nonspecific fluorescence from the forward (FSC) and side scatter (SSC) as parameters of cell size and granularity, respectively. Lymphocytes region was determined using SSC × FSC plot (G1) and monocytes region (G2) were selected based on their granularity and expression of CD14 (a). (b)represents the strategy for monocytes subsets classification through the expression of CD14 and CD16. Representative graph of one experiment.

The frequency of positive cells was analyzed by flow cytometry using the program flowjo in two regions. The lymphocyte region was determined using granularity (SSC) × size (FSC) plot. Monocytes were selected based on their granularity and expression of CD14 and CD16. Limits for the quadrant markers were always set based on negative populations and isotype controls. A representative density graph of one experiment showing lymphocyte and monocyte regions is shown in Figures 1(a) and 1(b), respectively.

### 2.5. Statistical Analyses

Statistical analyses were performed using the software GraphPad Prism (GraphPad Software, San Diego, CA). The frequency of positive cells was expressed as percentages and the intensity of expression as mean intensity fluorescence (MFI). The differences between means were assessed using Wilcoxon matched pairs test. Statistical significance was established at the 95% confidence interval.

## 3. Results

A total of 30 patients with cutaneous leishmaniasis were enrolled in this study, 17 were male and 13 were female, with a mean age of 29.1 ± 11.8 years (range 6–48 years). The majority of patients presented with a single lesion (80%) and the median lesions size was 130 mm² (IQR, 66.5–342; Table 1).

### 3.1. The Effect of the Addition of* S. mansoni *Antigens on Monocyte Phenotype and Expression of Co-Stimulatory Molecules

**Figure 2:**
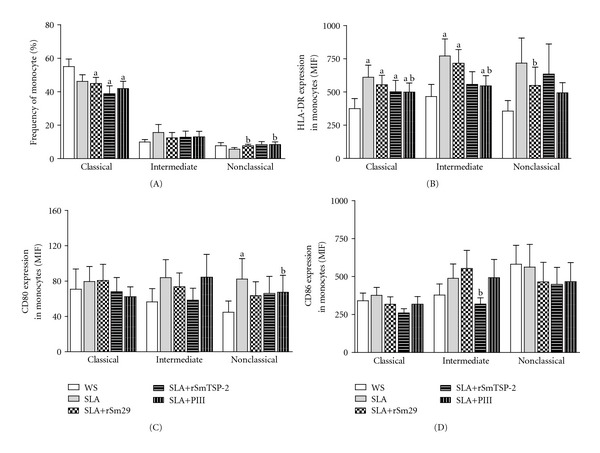
Subsets of monocytes and co-stimulatory molecules expression in monocytes of CL patients (*n* = 18) stimulated *in vitro* with SLA in the presence or absence of *S. mansoni* antigens rSm29 and rSmTSP-2 or with a fraction of *S. mansoni *soluble adult worm antigen (SWAP) PIII. Frequency of classical (CD14^++^CD16^−^), intermediate (CD14^++^CD16^+^) and nonclassical (CD14+CD16^++^), monocytes (A), and expression of HLA-DR, CD80, and CD86 in classical, intermediate and nonclassical monocytes of cutaneous leishmaniasis patients (B–D). Cells were stained for surface expression of CD14, CD16, HLA-DR, CD80, and CD86 using flow cytometry. ^a^ *P* < 0.05 cultures without stimulation (WS) versus SLA + *S. mansoni* antigens; ^b^ *P* < 0.05 SLA versus SLA + *S. mansoni* antigens (Wilcoxon matched pairs test).

We evaluated the frequency of different monocyte subsets (classical, intermediate, and nonclassical) and also the expression of co-stimulatory molecules in these monocytes after *in vitro* stimulation with SLA in the presence or absence of *S. mansoni* antigens. The addition of the antigens rSm29 and PIII to the cultures expanded the frequency of nonclassical (CD14^+^CD16^++^) (mean ± SEM = 7.4 ± 1.2% and 8.1 ± 1.9%, resp.) compared to cultures stimulated with SLA alone (5.7 ± 0.9%; *P* < 0.05.  Figure 2(A)). We also observed that the frequency of classical monocytes was higher in cultures without *S. mansoni* antigens (Figure 2(A)). Moreover, in the presence of PIII there was a reduction in the expression of HLA-DR in classical (CD14^++^CD16^−^) and intermediate (CD14^++^CD16^+^) monocytes (496 ± 72 MFI and 544 ± 78.6 MFI, resp.) compared to cultures stimulated with SLA alone (611 ± 91 MFI and 771 ± 128 MFI, respectively; *P* < 0.05.  Figure 2(B)). There was also a reduction in HLA-DR expression on CD14^+^CD16^++^ monocytes in the presence of rSm29 (547 ± 140.5 MFI) in comparison to SLA alone (718 ± 188.4 MFI; *P* < 0.05.  Figure 2(B)). Additionally, the expression of HLA-DR was higher in classical and intermediate monocytes in the presence of SLA or SLA plus *S. mansoni* antigens compared to nonstimulated cultures.

The expression of CD80 was also reduced in nonclassical monocytes compared to cultures stimulated with SLA. The addition of PIII to the cultures also resulted in reduced expression of the co-stimulatory molecule CD80 on nonclassical monocytes (61.2 ± 19.5 MFI) compared to cultures without *S. mansoni* antigens (82.3 ± 23.3 MFI; *P* < 0.05.  Figure 2(C)). Also a decrease in the expression of CD86 on intermediate monocytes from 562 ± 149.7 MFI to 447.8 ± 112.5 MFI was observed when rSmTSP-2 was added to the cultures (*P* < 0.05; Figure 2(D)). There was no significant difference in the levels of CD86 expression on monocytes in culture stimulated with SLA after the addition of rSm29 and PIII (Figure 2(D)). 

### 3.2. The Effect of the Addition of* S. mansoni *Antigens on T Cell Phenotype and Expression of Co-Stimulatory Molecules

**Figure 3:**
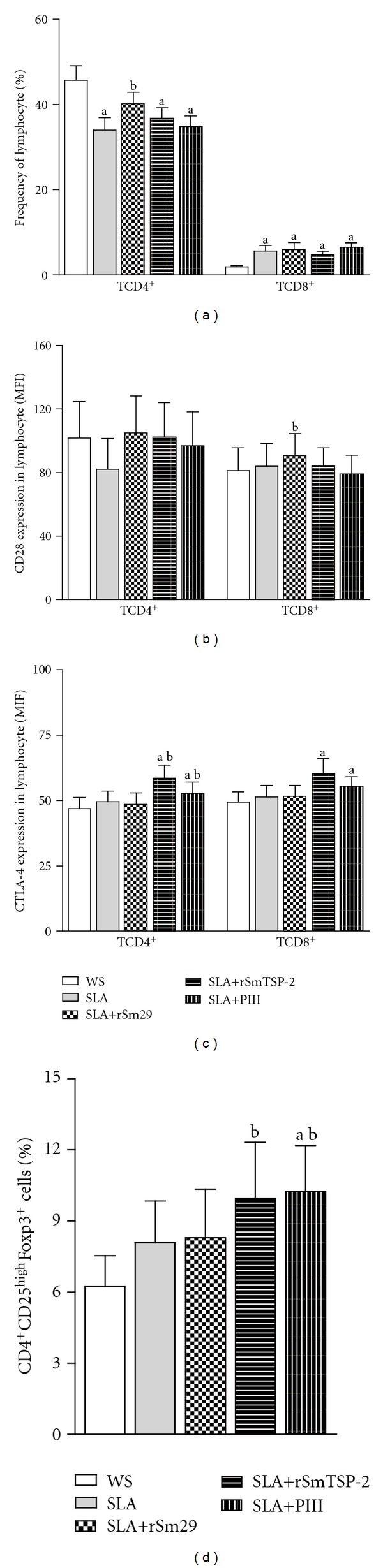
Phenotype of T cells and co-stimulatory molecules expression on T lymphocytes of CL patients (*n* = 17) stimulated *in vitro* with SLA in the presence or absence of *S. mansoni* antigens rSm29, rSmTSP-2 or with a fraction of *S. mansoni* soluble adult worm antigen (SWAP) PIII. Frequency of CD4^+^ and CD8^+^ T cells (A), expression of CD28 and CTLA-4 in CD4^+^ and CD8^+^ T cells (B and C, resp.) and the frequency of CD4^+^CD25^high^Foxp3^+^ T cells (*n* = 13) (D). Cells were stained for surface expression of CD4, CD8, CD25 and CD28, while the expression of CTLA-4 and Foxp3 were evaluated intracellularly using flow cytometry. ^a^ *P* < 0.05 cultures without stimulation (WS) versus SLA + *S. mansoni* antigens; ^b^ *P* < 0.05 SLA versus SLA + *S. mansoni* antigens (Wilcoxon matched pairs test).

PBMC of CL patients were incubated with SLA in the presence or absence of *S. mansoni *antigens and the phenotype of T cells were evaluated. The addition of rSm29 antigen to the cultures increased the frequency of CD4^+^ T cells (mean ± SEM = 40.8 ± 2.8%) in comparison to cultures with SLA alone (34.8 ± 2.8%, *P* < 0.05; Figure 3(A)). There was no significant difference in the frequency of CD4^+^ T cells after the addition of rSmTSP-2 or PIII antigens to the cultures (37.8 ± 2.7% and 35.8 ± 2.6%, resp.) compared to SLA alone (34.8 ± 2.8%; Figure 3(A)). Likewise, there was no significant variation in the frequency of CD8^+^ T cells by the presence of rSm29, rSmTSP-2, and PIII antigens (5.9 ± 1.2%, 4.6 ± 0.8%, and 6.2 ± 1.1%, resp.) to the cultures in relation to the cultures stimulated with SLA without* S. mansoni *antigens (5.7 ± 1.2%; Figure 3(A)). The frequency of CD4^+^ T cells was diminished by the presence of SLA and SLA plus rSmTSP-2 or PIII, while the frequency of CD8^+^ T cells was higher in the presence of SLA and SLA plus *S. mansoni* antigens compared to nonstimulated cultures (Figure 3(A)).

We also evaluated the expression of CD28^+^ on lymphocytes in cultures stimulated with SLA with or without the addition of *S. mansoni* antigens. The expression of CD28^+^ on CD8^+^ T cells was higher in cultures stimulated with rSm29 (91 ± 14 MFI, resp.) compared with SLA alone (84 ± 14 MFI; *P* < 0.05. Figure 3(B)). The addition of rSmTSP-2 and PIII antigens to the cultures did not alter significantly the expression of CD28 on CD4^+^ and CD8^+^ T cells (Figure 3(B)).

Regarding the expression of CTLA-4, rSmTSP-2 and PIII antigens were able to increase the expression of this molecule in CD4^+^ T cells (58 ± 5 MFI and 53 ± 4.6 MFI, resp.) in comparison with SLA alone (49.6 ± 4 MFI, respectively; *P* < 0.05. Figure 3(C)). The expression of CTLA-4 was also higher in CD4^+^ T cells and CD8^+^ T cells in cultures stimulated with SLA plus rSmTSP-2 or PIII compared to nonstimulated cultures (Figure 3(C)). Moreover, the addition of rSmTSP-2 and PIII antigens to the cultures expanded the frequency of CD4^+^CD25^high^Foxp3^+^ T cells (10 ± 2.4% and 10.3 ± 1.9%, resp.) compared to cultures with SLA in the absence of *S. mansoni* antigens (8.1 ± 1.8%; *P* < 0.05. Figure 3(D)). The frequency of CD4^+^CD25^high^Foxp3^+^ T cells was also higher in cultures stimulated with SLA plus PIII compared to nonstimulated cultures (Figure 3(D)).

## 4. Discussion

The Th1 immune response associated with macrophage activation and killing of *Leishmania* sp. is paradoxically related to the development of cutaneous and mucosal leishmaniasis. In an attempt to control parasite growth, activated macrophages release high levels of proinflammatory cytokines and nitrogen and oxygen reactive intermediates, leading to a tissue lesion.

A balance between the proinflammatory response characterized by the production of IFN-*γ* and TNF, and the regulatory response with the production of IL-10 has been observed in individuals exposed to the *Leishmania braziliensis *in endemic areas of leishmaniasis in Brazil. Those individuals do not develop the disease and they are designated as “subclinical” subjects [37]. The balanced immune response described in these individuals may be the key to achieving a harmless host-parasite relationship. 

 There are other evidences that IL-10 is capable of downmodulating the inflammatory response associated with human tegumentary leishmaniasis [4, 38–40]. However, mononuclear cells of mucosal leishmaniasis (ML) patients have a decreased ability to produce this cytokine and to respond to IL-10 after *in vitro* restimulation with *L. braziliensis *antigen [3].

Recently it has been shown that during chronic *Schistosoma mansoni* infection, cells from the innate immune response, such as monocytes and regulatory T cells produce high levels of IL-10 [24, 41–43]. Our group has performed studies in an attempt to identify *S. mansoni* antigens with regulatory properties that enable them to downregulate the inflammatory process associated with certain immune-mediated diseases. For instance, we have shown that the *S. mansoni* antigens rSm29, rSmTSP-2, and PIII induce IL-10 and IL-5 production by PBMC of cutaneous leishmaniasis patients and that they are able to control the *in vitro* inflammatory response in a group of patients, independent of the clinical features, such as number and size of lesions [8]. In this current study we extended the assessment of the potential of the antigens rSm29, rSmTSP-2, and PIII in modifying the immune response during cutaneous leishmaniasis. Specifically, we evaluated the impact of the addition of these antigens to the cell culture stimulated with *Leishmania* antigens on monocyte and lymphocyte profile and activation status. 

CD4^+^ T lymphocytes and monocytes are key cells in the protection against leishmaniasis; however they have been associated to the inflammatory process and tissue lesion in the cutaneous form of disease [20, 44–46]. In leishmaniasis and also in some others diseases the role of different subsets of monocytes in the pathology has been described [47]. For more than two decades, monocytes have been classified into classical (CD14^+^CD16^−^) and inflammatory, those which express the molecule CD16 and produce high levels of TNF-*α* [48]. The frequency of CD14^+^CD16^+^ in CL patients was found to be significantly higher compared to healthy controls and they were positively correlated with the lesion size [22]. 

Recently, a new nomenclature has been used to classify human blood monocytes into three subsets: classical (CD14^++^CD16^−^), intermediate (CD14^++^CD16^+^), and nonclassical (CD14^+^CD16^++^) [15]. The classical monocytes account for about 85% of the total monocytes and are characterized by the expression of IL-13R*α*1, IL-10, and RANTES and by the expression of genes associated with anti-apoptosis and wound healing properties. The intermediate monocytes represent 5% of monocytes, express high levels of HLA-DR and are associated with antigen processing and presentation to T cell and T cell activation. Nonclassical subset are characterized by a low expression of CD14 and high expression of CD16 (CD14^+^CD16^++^). They represent 10% of human monocytes and studies have shown that they express genes associated with cytoskeletal rearrangement which account for their highly mobility and for the patrolling behavior *in vivo* [16, 47]. 

Since the roles of the different monocyte subsets in cutaneous leishmaniasis remain unclear, we decided to evaluate not only their frequency in leishmaniasis patients, but also whether the addition of *S. mansoni* antigens would alter the profile of these cells in an *in vitro* study. 

The addition of the antigens rSm29 and PIII to the PBMC cultures stimulated with SLA increased the frequency of nonclassical monocytes. This was not expected as a cell with high mobility and migratory capacity [16, 47], they may contribute to *Leishmania* metastasis and consequent development of more severe forms of the disease, such as the mucosal and disseminated forms.

We found, however, that the *S. mansoni* antigens used in this study, particularly PIII, diminished the expression of HLA-DR as well as the expression of the co-stimulatory molecules B7.1 and B7.2 on different monocyte subsets. A downmodulation of monocyte activation is desirable in leishmaniasis and may result in a reduced inflammatory process. 

In this study, we also evaluated the expression of co-stimulatory molecules in T cells. We observed a significant increase in the frequency of CD4^+^ T cells by the presence of the rSm29 antigen in the cultures. The addition of rSmTSP-2 and PIII antigens to the cultures stimulated with SLA increased the expression of CTLA-4 in CD4^+^ T cells and also expanded the frequency of CD4^+^CD25^high^Foxp3^+^ T cells. We previously showed that *S. mansoni *infection expands CD4^+^ T cell population of the regulatory profile in *S. mansoni*-infected asthmatic individuals [24]. In such allergic disease, *S. mansoni* antigens downmodulate the Th2 exacerbated inflammatory response *in vitro* by inducing the production of IL-10 and the expression of the regulatory molecules, CTLA-4 in T lymphocytes [9, 24, 43]. In a murine model of ovalbumin-induced asthma, inhibition of lung inflammatory process, by *S. mansoni* eggs or by the *S. mansoni* antigens, Sm22.6, PIII, and rSm29 were associated with an increase in the number of CD4^+^CD25^+^Foxp3^+^ T cells and high levels of IL-10 production [49, 50]. 

T cell-mediated immunity is fundamental to host protection against *Leishmania* sp. and the activation of T cells depends upon signals from the interaction between the co-stimulatory molecules CD28 to its ligands B7.1 and B7.2 on antigen presenting cells (APCs) [23]. We evaluated the expression of CD28 on lymphocytes in cultures stimulated with SLA with or without the addition of *S. mansoni* antigens and found that the expression of this molecule on CD8^+^ T cells was higher in cultures stimulated with rSm29 compared with SLA alone. These results suggest that the CD8^+^ T cells became more activated in the presence of rSm29. There was, however, an increased expression of CTLA-4 in CD4^+^ T cells, a molecule which is expressed to inhibit the T cell activation when rSmTSP-2 and PIII were added to the cultures. *In vitro* study has shown that the addition of CTLA-4-Ig to block CD28-B7 interaction in CL patients, PBMC cultures stimulated with* Leishmania* antigen led to a downmodulation of IFN-*γ*, IL-10, and TNF secretion [27]. Our findings suggest that the CD4^+^ T cells were downmodulated by the presence of the rSmTSP-2 and PIII. These antigens were also able to increase the frequency of the CD4^+^CD25^high^Foxp3^+^ regulatory T cells.

In a survey performed by O'Neal et al. [51], the prevalence of *S. mansoni* infection in cutaneous leishmaniasis patients from the endemic area of Corte de Pedra, Bahia, Brazil was 16.7% [51]. The authors also showed that coinfected individuals tended to have small lesion size, however in the univariated model, the presence of helminth coinfection was associated with delayed lesion healing. When they evaluated infection with *S. mansoni* and *Strongyloides stercoralis* there was no difference in lesion healing compared to patients infected with geohelminths such as *Ascaris lumbricoides*, *Ancylostoma duodenale,* and *Trichuris trichiura*.

Studies conducted by the same group in the endemic area of leishmaniasis in Brazil demonstrated that the use of immunomodulatory agents such as granulocyte/macrophage colony stimulating factor (GM-CSF) and pentoxifylline, an inhibitor of TNF production, can lead to faster healing time and higher cure rate in cutaneous leishmaniasis [52–55]. It has also been reported that intralesional injections of GM-CSF reduce healing time of CL ulcers by 50%. Moreover, Santos et al. [53] showed that GM-CSF applied locally in low doses as an adjuvant to pentavalent antimonial therapy, significantly decreases the healing time of CL ulcers, reducing the dose of antimony administered and/or the duration of antimonial therapy. These studies indicate that down-modulation of the inflammatory response in CL patients is associated with lesion cure and give support to the idea that inhibition of the strong inflammatory response early after *Leishmania* infection could avert tissue damage. 

In this context, the data presented herein suggest that *S. mansoni* antigens are capable of downregulating monocyte and T lymphocyte response to the *Leishmania* antigen *in vitro*, possibly through mechanisms that involve negative signaling by CTLA-4 and the action of regulatory T cells. This knowledge could contribute to the development of future strategies to prevent and treat cutaneous and mucosal leishmaniasis.

## Figures and Tables

**Table 1:** Demographic characteristics of the cutaneous leishmaniasis patients included in the study.

Characteristics (*n* = 30)	Values
Age, mean ± SD, years	29.1 ± 11.8
Sex, female/male	13/17
N° of lesion	
1, no. (%)	24 (80.0)
≥2, no. (%)	06 (20.0)
Size of lesion, median mm² (IQR)	130 (66.5–342)

IQR: interquartile range; SD: standard deviation.
